# The characteristics and risk factors of fatal falls among adults aged 60 and above in Southwest China

**DOI:** 10.1038/s41598-024-54265-9

**Published:** 2024-03-25

**Authors:** Rui Deng, Benyan Li, Mingfang Qin, Xinyi Yu, Junli Sun, Feng Jiao, Yuan Huang

**Affiliations:** 1 School of Public Health, Kunming Medical University, Kunming, 650500 China; 2 Department of Health Insurance, The Second Affiliated Hospital of Kunming Medical University, Kunming, 650106 China; 3 Yunnan Institute of Endemic Diseases Control & Prevention, Dali, 671000 China; 4 School of Public Health, Capital Medical University, Beijing, 100069 China; 5 Department of Acute Infectious Diseases and Immunization Program, Mengzi Center for Diseases Control and Prevention, Mengzi, 661100 China

**Keywords:** Public health, Quality of life, Health care, Risk factors

## Abstract

Falls constitute a leading cause of unintentional injury deaths among older adults. This study aimed to examine the comprehensive characteristics of fatal falls among older individuals in Yunnan Province, China, to highlight the challenges faced in elderly care. A total of 22,798 accidental fall-related deaths were extracted from China's National Disease Surveillance Points System aged 60 and above between 2015 and 2019. Quantitative and textual data were analyzed to assess the incidence rates of initiating factors, locations, symptoms, and overall survival (OS) outcomes after falling. Hypertension emerged as the most significant intrinsic factor, especially among individuals aged between 70 and 79, female older adults, and urban residents (*P* < 0.001). Home was identified as the most common location where fatal falls occurred (61.19%). The head was the most commonly injured body region (58.75%). The median of OS for all fatal falls was 2 days (0.13, 30), of which deaths occurred within 24 h [9287 (49.36%)]. There were instances where timely discovery after falling did not occur in 625 cases; their median of OS was significantly shorter compared to those discovered promptly after falling (*P* < 0.001). Targeted interventions focusing on fall prevention and post-fall care are equally crucial for the well-being of older adults.

## Introduction

Representing a pervasive unintentional injury, falls among older adults persist as a global challenge in both elderly care and public health. The rates of falls among older adults in different settings have been extensively studied, revealing significant variations. However, it is crucial to acknowledge that unpredictable and unforeseen falls in the senior population can result in severe trauma, hospitalizations, disabilities, and even mortality^1^. Fall-related injuries impose an increased risk of financial strain and caregiving burden on older adults and their families, potentially leading to higher poverty rates^2^. World Health Organization (WHO) reports a global annual incidence of approximately 684 000 fatal falls, with 80% of cases concentrated in low- and middle-income countries, especially within the Western Pacific and South East Asia countries^3^. Given that adults aged 60 years and above exhibit the highest mortality rates associated with falls, it is evident that falls constitute a primary cause of unintentional injury deaths within the seniors^3^. In China specifically, fatal falls have manifested substantial health loss and significant social costs. A meta-analysis conducted between 2001 and 2019 delineated an estimated incidence rate of falls among Chinese adults aged ≥ 60 years residing in communities at 14.3% (*95% CI*: 11.9–17.0)^4^. The escalation in fall-related deaths among Chinese individuals aged ≥ 60 years is noteworthy, increasing from 25 000 in 1999 to 107 000 in 2019, with corresponding deaths rates rising significantly from 25.19 per 100 000 persons in 1999 to 39.19 per 100 000 persons in 2019. This translates into an average daily toll of 276 elderly fatalities due to falls in China alone, highlighting a more than twofold increase (> 200%) in disease burden caused by falls among the older adults over three decades^5^.

During the recent past, numerous countries have witnessed a rapid increase in their aging populations. As of 2021, the global elderly population has surpassed one billion, accounting for 13.5% of the global population^6^. China, in particular, has experienced an accelerating trend in population aging since the 1990s. According to the seventh national census, the proportion of individuals aged 60 and above in China has risen from 13.3% in 2010 to 18.7% in 2020^7^. This underscores China’s standing as one of the most rapidly aging population globally emphasizing healthy aging as a central discourse and priority within its national strategic development agenda. To address this shift comprehensively, China established a long-term commitment through the issuance of the National Medium—and Long-term Plan for Actively Coping with Population Aging in 2019. This plan aims to provide an extensive range of high-quality healthcare services for older adults including health education, preventive care measures, disease diagnosis and treatment options, rehabilitation nursing facilities as well as long-term care and hospice care. This proactive approach aligns with the Healthy China 2030 initiative which strives towards achieving universal health coverage by offering comprehensive life-cycle health services. Improving elderly care quality has been identified as a promising aspect outlined within China’s “14th Five-year Plan” for national economic and social development.

While a body of empirical research has demonstrated the global magnitude of falls, encompassing their prevalence and frequency, associated risk or protective factors, subsequent mortality, and disease burden, as well as the effectiveness of various preventive measures^1,5,8–11^, existing studies have primarily focused on the elevated risk of falls among older adults due to multiple factors such as functional limitations, health conditions, vision impairments, medication usage and depression^10,12^. However, there is limited literature available on the fatality aspects of falls including how they become deadly and the time interval between a fall and death. Additionally, there is a scarcity of insights into identifying senior individuals who are at high risk for fatal falls. Existing analyses on fall-related mortality predominantly rely on quantitative data from national statistic systems or cross-sectional samples, which, though providing numerical indicators, lack the sufficient details required to comprehensively understand fatal falls. In addition to examining fall-related mortality and their distribution by sex and age^11^, three aspects related to fatal falls warrant further exploration: preconditions leading up to a fall event, injuries sustained during a fall, and post-fall care.

In this article, we extensively integrated quantitative and textual data extensively from China's National Disease Surveillance Points System (DSPs), managed by the Center for Disease Control and Prevention (CDC). The objective is to comprehensively examine the overall characteristics of fatal falls among older adults aged ≥ 60 years, including contributing factors, locations of occurrence, resulting injuries, and post-fall care received. By utilizing Yunnan province as a representative example, we meticulously analyze 22,798 fall-related deaths in adults aged 60 and above between 2015 and 2019. Through highlighting these incidents, our study aims to shed light on current challenges faced in elderly care while proposing strategies to prevent premature mortality. We firmly believe that an extended life expectancy signifies collective progress in economic development, social well-being, and health advancements. Longevity should be pursued as a desirable life course wherein older individuals can live longer with functional ability and dignity despite age-related physiological changes affecting hearing, vision, and mobility^6,12^.

## Methods

### Study setting

Yunnan, a Chinese province located in the southwestern frontier of the country, boasts remarkable biodiversity and an extensive reserve of natural resources. As the fifth largest province in China, Yunnan is home to the highest number of ethnic groups in the country, accounting for 33.1% of its total population (47.2 million in 2020). Despite being one of the less-developed provinces in China, Yunnan's Gross Domestic Product (GDP) reached RMB 2.5 trillion (US$384 billion) in 2020—amidst the pandemic year—with a notable year-on-year growth rate of 4.0 percent that surpassed the national average at 2.3 percent. Given its strategic economic and geographical position, Yunnan plays a pivotal role within China's Southwest Economic Circle city clusters and serves as an essential gateway to South and Southwest Asia regions. Alongside rapid socio-economic development over recent decades, Yunnan Province accommodated a demographic of 7.0 million older individuals (aged 60 or above) in 2020, constituting approximately 14.9% of its total population.

### Study design and data sources

A cross-sectional study was conducted to investigate the characteristics and risk factors associated with fatal falls, specifically unintentional falls resulting in death that were neither self-inflicted nor related to criminal activities. Fall-related deaths among adults aged 60 and above in Yunnan Province between 2015 and 2019, as defined by International Classification of Diseases, 10th Version (ICD-10) codes W00-19, were selected from the DSPs system which records individual-level death data for all residents within the region. Quantitative data on demographic characteristics of individuals who died from unintentional falls, contributing factors and locations of fatal falls, and overall survival (OS) after falling were extracted. Textual data including descriptions of initiating event, symptoms, and treatment process for each fatal fall were also analyzed.

### Statistical analysis

In order to gain a comprehensive understanding of the circumstances surrounding fatal falls, we employed the Who, What, Where, Why, and How (4W1H) Framework^13^, encompassing five key questions: (1) why did a fatal fall happen, (2) where did a fatal fall take place, (3) what were the consequences of a fatal fall, (4) how long did it take to die after a fatal fall, and (5) who were the high-risk group of fatal falls. By delineating both initiating events (contributing factors) and their corresponding locations, we aimed to elucidate the underlying reasons and the contexts of these fatal falls occurred. The initiating events were further categorized into intrinsic contributing factors including medical conditions, impaired mobility and vision, medication misuse, and alcohol use; while extrinsic comprised environmental hazards, occupational risks associated with work environments or activities undertaken therein, along with interpersonal conflicts. Moreover, based on categorization criteria encompassing domestic settings alongside public spaces and working places respectively, we sought to comprehend how injuries sustained during falls culminated in fatalities by examining not only physical trauma but also subsequent complications such as infections and loss of functional abilities.

Meanwhile, fatal falls were categorized based on the fall height (falls on same levels and falls from a height) and the injured body part. The OS of different categories was described using median, interquartile range, and proportions over five designated time periods (0–24 h, one day-7 days, 8 days-30 days, one month-12 months, and > 1 year). Furthermore, unmet healthcare needs were assessed by comparing the proportions and OS of older adults who received post-fall treatment with those who did not receive treatment or remained undiscovered. Subgroup analyses stratified by gender, age, and living places were conducted using Pearson's chi-square test or the Kruskal–Wallis test to identify high-risk populations for fatal falls. All statistical analyses were performed using Stata 17.0 at a significance level set of 5%.

### Textual data analysis

The process leading up to death in fatal falls was examined through thematic analysis of textual data from death records. A Java-based software (No. 2021SR0597365) was developed to facilitate keyword extraction, coding, and grouping within a framework comprising 47 themes, achieving a pilot assessment accuracy of 90% with 6000 text entries. Extracted information from the textual data enabled identification of various themes and their interrelationships, thereby enhancing understanding of fatal falls among adults aged 60 and above in Southwest China.

### Ethics approval and consent statement

The research protocol was approved by the Institutional Review Board and Ethics Committee of Kunming Medical University (KMMU2022MEC027). It was conducted in accordance with the ethical standards outlined in the Declaration of Helsinki. All methods were performed following the relevant guidelines and regulations. The research was conducted using a de-identified secondary dataset, which had been anonymized prior to its receipt by the research team. Informed consent was obtained from respondents by Yunnan CDC before data collection. Therefore, the Ethics Committee of Kunming Medical University waived the requirement for obtaining informed consent for the research team.

## Results

Between 2015 and 2019, a total of 22 798 deaths resulting from accidental falls among adults aged 60 and above in Yunnan Province were included in this study. Table 1 provides an overview of the contributing factors which initiated these fatal falls, elucidating that intrinsic factors accounted for 18.92% (n = 4 313) of fatal falls, while extrinsic factors contributed to 9.16% (n = 2 089), and the causes remained indeterminate in 74.53% (n = 16 992) of cases. Hypertension emerged as the most significant factor with a prevalence rate of 11.33% (n = 2 583). This problem was particularly pronounced among individuals aged between 70 and 79 years (*χ*^*2*^ = 41.699, *P* < 0.001), female older adults (*χ*^*2*^ = 19.945, *P* < 0.001) and urban residents (*χ*^*2*^ = 64.806, *P* < 0.001) compared to other demographic groups analyzed in this study. Although work-related activities were not identified as the leading cause of fatal falls among older adults examined in this study, they exhibited greater prominence among individuals under the age of 70 (*χ*^*2*^ = 609.185, *P* < 0.001), male older adults (*χ*^*2*^ = 122.561, *P* < 0.001) and rural residents (*χ*^*2*^ = 81.670, *P* < 0.001) when compared with other demographic subgroups.

**Table 1:** The main contributing factors of 22,798 fatal falls among adults aged 60 and above in Yunnan between 2015 and 2019 (N, %).

Contributing factors	All cases	Age			Gender		Places	
		60–69 years	70–79 years	≥ 80 years	Male cases	Female cases	Urban cases	Rural cases
Total (N)	22,798	4664	6691	11,443	11,746	11,052	4864	17,931
Intrinsic factors	4313 (18.92)	796 (17.07)	1405 (21.00)	2112 (18.46)	2120 (18.05)	2193 (19.84)	1101 (22.64)	3212 (17.91)
Medical conditions	4071 (17.86)	652 (13.98)	1346 (20.12)	2073 (18.12)	1919 (16.34)	2152 (19.47)	1066 (21.92)	3005 (16.76)
Cardiac	271 (1.19)	33 (0.71)	82 (1.23)	156 (1.36)	132 (1.12)	139 (1.26)	99 (2.04)	172 (0.96)
Hypertension	2583 (11.33)	429 (9.20)	875 (13.08)	1279 (11.18)	1224 (10.42)	1359 (12.30)	709 (14.58)	1874 (10.45)
Neurological	437 (1.92)	77 (1.65)	148 (2.21)	212 (1.85)	207 (1.76)	230 (2.08)	107 (2.20)	330 (1.84)
Other diseases	1588 (6.79)	1229 (4.91)	525 (7.85)	834 (7.29)	722 (6.15)	866 (7.84)	438 (9.00)	1150 (6.41)
Poor mobility and vision	187 (0.82)	19 (0.41)	53 (0.79)	115 (1.00)	75 (0.64)	112 (1.01)	31 (0.64)	156 (0.87)
Medication misuse	72 (0.32)	9 (0.19)	24 (0.36)	39 (0.34)	33 (0.28)	39 (0.35)	27 (0.56)	45 (0.25)
Alcohol use	216 (0.95)	139 (2.98)	52 (0.78)	25 (0.22)	200 (1.70)	16 (0.14)	34 (0.70)	182 (1.02)
Extrinsic factors	2089 (9.16)	707 (5.16)	598 (8.94)	784 (6.85)	1285 (10.94)	804 (7.27)	341 (7.01)	1747 (9.74)
Environmental hazards	1235 (5.42)	267 (5.72)	336 (5.02)	632 (5.52)	687 (5.85)	548 (4.96)	262 (5.39)	973 (5.43)
Work and occupations	848 (3.72)	438 (9.39)	263 (3.93)	147 (1.28)	595 (5.07)	253 (2.29)	75 (1.54)	772 (4.31)
Interpersonal conflicts	29 (0.13)	12 (0.26)	8 (0.12)	9 (0.08)	19 (0.16)	10 (0.09)	6 (0.12)	23 (0.13)
Other unclear factors	16992 (74.53)	3370 (72.26)	4876 (72.87)	8746 (76.43)	8707 (74.13)	8258 (74.96)	3546 (72.90)	13,444 (74.98)

The locations where the fatal falls occurred among older adults are presented in Table 2. Home was identified as the most prevalent setting for these incidents, accounting for 61.19% of cases, followed by the workplace (6.09%) and public areas (5.86%). Elevated rates of fatal falls at home were observed among individuals aged over 80 years (67.75% vs. 59.11% and 48.09%, *P* < 0.001), females (65.81% vs. 56.85%, *P* < 0.001) and urban residents (64.68% vs 60.25%, *P* < 0.001). Conversely, a greater incidence of fatal falls in occupational settings was found among seniors under the age of 70 (14.41% vs. 6.82% and 2.28%, *P* < 0.001), males (8.29% vs. 3.75%, *P* < 0.001) and rural residents (6.87% vs 3.23%, *P* < 0.001).

**Table 2:** The Locations where 22,798 Fatal Falls Occurred among adults aged 60 and above in Yunnan between 2015 and 2019 (N, %).

Locations	All cases	Age groups			Gender		Places	
		60–69 years	70–79 years	≥ 80 years	Male cases	Female cases	Urban cases	Rural cases
Total (N)	22,798	4664	6691	11,443	11,746	11,052	4864	17,931
Home	13,951 (61.19)	2243 (48.09)	3955 (59.11)	7753 (67.75)	6678 (56.85)	7273 (65.81)	3146 (64.68)	10,803 (60.25)
Bathroom	341 (1.50)	57 (1.22)	113 (1.69)	171 (1.49)	186 (1.58)	155 (1.40)	77 (1.58)	264 (1.47)
From bed	441 (1.93)	51 (1.09)	117 (1.75)	273 (2.39)	198 (1.69)	243 (2.20)	105 (2.16)	336 (1.87)
From chair	158 (0.69)	30 (0.64)	40 (0.60)	88 (0.77)	84 (0.72)	74 (0.67)	32 (0.66)	126 (0.70)
Stairs and steps	4492 (19.70)	889 (19.06)	1408 (21.04)	2195 (19.18)	2293 (19.52)	2199 (19.90)	674 (13.86)	3817 (21.29)
Public areas	1336 (5.86)	315 (6.75)	416 (6.22)	605 (5.29)	711 (6.05)	625 (5.66)	312 (6.41)	1024 (5.71)
Community	844 (3.70)	174 (3.73)	260 (3.89)	410 (3.58)	418 (3.56)	426 (3.85)	205 (4.21)	639 (3.56)
Stairs and steps	214 (0.94)	48 (1.03)	72 (1.08)	94 (0.82)	117 (1.00)	97 (0.88)	40 (0.82)	174 (0.97)
Roads	412 (1.81)	121 (2.59)	127 (1.90)	164 (1.43)	242 (2.06)	170 (1.54)	91 (1.87)	321 (1.79)
Sport areas	65 (0.29)	24 (0.51)	21 (0.31)	20 (0.17)	38 (0.32)	27 (0.24)	7 (0.14)	58 (0.32)
Other public areas	62 (0.27)	16 (0.34)	24 (0.36)	22 (0.19)	43 (0.37)	19 (0.17)	15 (0.31)	47 (0.26)
Working place	1389 (6.09)	672 (14.41)	456 (6.82)	261 (2.28)	974 (8.29)	415 (3.75)	157 (3.23)	1231 (6.87)
Agriculture	1024 (4.57)	451 (9.67)	376 (5.62)	215 (1.88)	696 (5.93)	346 (3.13)	105 (2.16)	937 (5.23)
Industry	313 (1.37)	206 (4.42)	72 (1.08)	35 (0.31)	257 (2.19)	56 (0.51)	44 (0.90)	268 (1.49)
Services	39 (0.17)	18 (0.39)	9 (0.13)	12 (0.10)	25 (0.21)	14 (0.13)	9 (0.19)	30 (0.17)

Table 3 presents a comprehensive overview of fall-related injuries and associated physical consequences. The analysis reveals that physical injuries constituted the largest proportion (79.67%), followed by infections (13.73%) and loss of functionality (4.58%). A detailed examination of the data revealed that head trauma emerged as the most severely affected body region (58.75%), with femur fracture ranking second in terms of prevalence (12.16%). Distinct patterns were observed among different demographic groups: individuals under 70 years old, male older individuals, and rural residents exhibited a higher likelihood of experiencing head trauma, while those aged 80 and above, female older individuals and urban residents displayed an increased propensity for femur fracture (*χ*^*2*^ = 394.297, *P* < 0.001). Infections following falls were more prevalent among people aged 80 and above, female older individuals, and urban residents, with lower respiratory tract infections being particularly notable in this context. Similarly, older adults aged 80 and above along with female older individuals demonstrated a heightened susceptibility to functional decline subsequent to falling.

**Table 3:** The injuries and other physical consequences of 22,798 fatal falls among adults aged 60 and above in Yunnan between 2015 and 2019 (N, %).

Physical consequences	All cases	Age groups			Gender		Places	
		60–69 years	70–79 years	≥ 80 years	Male cases	Female cases	Urban cases	Rural cases
Total (N)	22,798	4664	6691	11,443	11,746	11,052	4864	17,931
Injuries	18,163 (79.67)	3805 (81.58)	5414 (80.91)	8944 (78.16)	9498 (80.86)	8665 (78.40)	3912 (80.43)	14,248 (79.46)
Head trauma	13,393 (58.75)	3383 (72.53)	4348 (64.98)	5662 (49.48)	7638 (65.03)	5755 (52.07)	2539 (52.20)	10,851 (60.52)
Femur fracture	2773 (12.16)	134 (2.87)	576 (8.61)	2063 (18.03)	1015 (8.64)	1758 (15.91)	814 (16.74)	1959 (10.93)
Spine, pelvic, or hip fracture	880 (3.86)	65 (1.39)	185 (2.76)	630 (5.51)	300 (2.55)	580 (5.25)	274 (5.63)	606 (3.38)
Rib fracture	253 (1.11)	54 (1.16)	79 (1.18)	120 (1.05)	142 (1.21)	111 (1.00)	51 (1.05)	202 (1.13)
Other bone fracture	1327 (5.82)	227 (4.87)	343 (5.13)	757 (6.62)	577 (4.91)	750 (6.79)	345 (7.09)	982 (5.48)
Visceral injury	184 (0.81)	61 (1.31)	64 (0.96)	59 (0.52)	117 (1.00)	67 (0.61)	34 (0.70)	150 (0.84)
Infections	3130 (13.73)	186 (3.99)	673 (10.06)	2271 (19.85)	1175 (10.00)	1955 (17.69)	902 (18.54)	2228 (12.43)
Decubitus ulcer infection	885 (3.88)	46 (0.99)	201 (3.00)	638 (5.58)	296 (2.52)	589 (5.33)	216 (4.44)	669 (3.73)
Lower respiratory tract infection	2144 (9.40)	112 (2.40)	454 (6.79)	1578 (13.79)	841 (7.16)	1303 (11.79)	693 (14.25)	1451 (8.09)
Other infections	198 (0.87)	33 (0.71)	47 (0.70)	118 (1.03)	77 (0.66)	121 (1.09)	27 (0.56)	171 (0.95)
Loss of ability	1044 (4.58)	98 (2.10)	246 (3.68)	700 (6.12)	389 (3.31)	655 (5.93)	207 (4.26)	837 (4.67)
Other consequences	983 (4.31)	151 (3.24)	276 (4.12)	55 (4.86)	470 (4.00)	513 (4.64)	355 (7.30)	627 (3.50)

Among the 22 798 fall-related fatalities, a total of 18 815 cases were reported with available OS data. As presented in Table 4, the median OS for all fatal falls was found to be 2 (0.13, 30) days. Notably, deaths occurring within the first 24 h accounted for the majority [9287 (49.36%)], succeeded by those between 1 and 7 days [2958 (15.72%)], and those within the interval from 1 to 12 months [2895 (15.39)]. Subgroup analysis revealed that individuals aged between 60 and 69 years exhibited an exceptionally short median OS of only 0.25 (0.04, 2) days compared to those aged ≥ 80 years old (*P* < 0.001). Furthermore, males demonstrated a shorter median OS [1 (0.08, 10)] than females [3 (0.21, 90)](*P* < 0.001), while rural residents displayed a shorter median OS [1 (0.08,28)] compared to their urban counterparts [6 (0.29, 77)](*P* < 0.001). In terms of fall location, falling from one level to another resulted in a shorter median OS [1 (0.08, 8)] than falling on the same level [3 (0.21, 60)] (*P* < 0.001). The shortest median OS was observed among individuals with visceral injury [0.42 (0.08, 3)], followed by head trauma [1 (0.08, 3)], and rib injury [8 (1, 30)].

**Table 4:** The overall survivals of 18,815 fatal falls among adults aged 60 and above in Yunnan between 2015 and 2019 (OS, Median and Inter Quartile Range, in Days).

Fatal falls		All cases	Age groups			Gender		Places	
			60–69 years	70–79 years	≥ 80 years	Male cases	Female cases	Urban cases	Rural cases
All fatal falls (N = 18,815)	OS	2 (0.13, 30)	0.25 (0.04, 2)	1 (0.08, 10)	6 (1, 90)	1 (0.08, 10)	3 (0.21, 90)	6 (0.29, 77)	1 (0.08, 28)
0–24 h	N, %	9287 (49.36)	2654 (71.31)	3053 (56.34)	3580 (37.01)	5434 (56.60)	3853 (41.82)	1501 (37.81)	7784 (52.44)
One day-7 days	N, %	2958 (15.72)	507 (13.62)	888 (1.39)	1563 (16.16)	1558 (16.23)	1400 (15.19)	640 (16.12)	2318 (15.62)
8 days-30 days	N, %	2290 (12.17)	253 (6.80)	545 (10.06)	1492 (15.42)	1073 (11.18)	1217 (13.21)	631 (15.89)	1659 (11.18)
1 month-12 months	N, %	2895 (15.39)	194 (5.21)	605 (11.16)	2096 (21.67)	1073 (11.18)	1822 (19.77)	824 (20.76)	2071 (13.95)
> 1 year	N, %	1385 (7.36)	114 (3.06)	328 (6.05)	943 (9.75)	463 (4.82)	922 (10.01)	374 (9.42)	1011 (6.81)
Falls on the same level (N = 12,005)	OS	3 (0.21, 60)	1 (0.08, 3)	1 (0.13, 20)	10 (1, 90)	1 (0.13, 20)	7 (1, 90)	7 (1, 90)	2 (0.17, 30)
0–24 h	N, %	5252(43.75)	1283 (66.55)	1732 (52.00)	2237 (33.16)	2955 (51.13)	2297 (36.89)	1010 (34.41)	4241 (46.76)
One day-7 days	N, %	1902 (15.84)	294 (15.25)	551 (16.54)	1057 (15.67)	957 (16.56)	945 (15.18)	474 (16.15)	1428 (15.75)
8 days-30 days	N, %	1649 (13.74)	146 (7.57)	375 (11.26)	1128 (16.72)	747 (12.93)	902 (14.49)	488 (16.63)	1161 (12.80)
1 month-12 months	N, %	2220 (18.49)	138 (7.16)	457 (13.72)	1625 (24.09)	796 (13.77)	1424 (22.87)	674 (22.96)	1546 (17.05)
> 1 year	N, %	982 (8.18)	67 (3.48)	216 (6.48)	699 (10.36)	324 (5.61)	658 (10.57)	289 (9.85)	693 (7.64)
Falls from one level to another (N = 6810)	OS	1 (0.08,8)	0.13 (0.04,1)	1 (0.08,4)	2 (0.13,30)	1 (0.08,4)	1 (0.08,30)	2 (0.13,30)	1 (0.08,7)
0–24 h	N, %	4035(59.25)	1371 (76.42)	1321 (63.27)	1343 (45.87)	2479 (64.86)	1556 (52.07)	491 (47.44)	3543 (61.36)
One day-7 days	N, %	1056 (15.51)	213 (11.87)	337 (16.14)	506 (17.28)	601 (15.72)	455 (15.23)	166 (16.04)	890 (15.41)
8 days-30 days	N, %	641 (9.41)	107 (5.96)	170 (8.14)	364 (12.43)	326 (8.53)	315 (10.54)	143 (13.82)	498 (8.62)
1 month-12 months	N, %	675 (9.91)	56 (3.12)	148 (7.09)	471 (16.09)	277 (7.25)	398 (13.32)	150 (14.49)	525 (9.09)
> 1 year	N, %	403 (5.92)	47 (2.62)	112 (5.36)	244 (8.33)	139 (3.64)	264 (8.84)	85 (8.21)	318 (5.51)
Severe injuries from falls (N = 15,361)	OS	1 (0.13, 21)	0.29 (0.04, 2)	1 (0.08, 7)	4 (0.5, 60)	1 (0.08, 7)	3 (0.21, 60)	4 (0.25, 60)	1 (0.08, 15)
Head trauma (N = 11,127)	OS	1 (0.08, 3)	0.21 (0.04, 1)	0.83 (0.08, 2)	1 (0.08, 3)	1 (0.08, 2)	1 (0.08, 3)	1 (0.08, 4)	1 (0.08, 2)
Femur fracture (N = 2511)	OS	90 (30, 365)	150 (17, 730)	90 (22, 365)	90 (30, 365)	90 (20, 365)	150 (30, 365)	90 (30, 365)	120 (30, 35)
Spine, pelvic, or hip fracture (N = 799)	OS	90 (30, 365)	90 (1, 730)	90 (20, 365)	90 (30, 365)	90 (30, 365)	90 (24, 365)	150 (30, 365)	90 (20, 365)
Rib fracture (N = 223)	OS	8 (1, 30)	2 (0.13, 10)	7 (1, 30)	13 (3, 40)	6.5 (1, 20)	15 (3, 43)	15 (3, 60)	7 (1, 30)
Other bone fracture (N = 1106)	OS	21.5 (1, 180)	1.17 (0.13, 30)	10 (0.25, 120)	36 (7, 330)	12 (0.25, 90)	30 (3, 300)	46 (7, 365)	15 (1, 150)
Visceral injury (N = 149)	OS	0.42 (0.08, 3)	0.13 (0.04, 1)	0.42 (0.08, 1)	3 (0.21, 22)	0.21 (0.04, 1)	1 (0.13, 4)	0.23 (0.04, 3)	0.5 (0.08, 3)

Table 5 further illustrates the disparities in OS between individuals who were timely found after falling and those who were not, as well as between those who received medical care after falling and those who did not. Among the 625 (3.32%) cases that were not timely found after falling, their median OS [0.17 (0.04, 1)] was significantly shorter than those of individuals who were timely found [2 (0.13, 30)] (*P* < 0.001). Subgroup analysis unveiled that individuals aged 60–69 years, males, and rural residents had a higher likelihood of being undiscovered after falling compared to other demographic groups (*P* < 0.001). Regarding medical care, among the 5649 (30.02%) cases that did not receive care after falling, their median OS [1 (0.04, 4)] was significantly shorter than that of individuals who received care [3 (0.25, 60)]. Subgroup analysis also showed that elderly individuals aged 60–69 years old, male, and rural residents had a higher likelihood of remaining untreated after falling.

**Table 5:** The Unmet health care needs of 18,815 fatal falls among adults aged 60 and above in Yunnan between 2015 and 2019 (OS, Median and Inter Quartile Range, in Days).

Unmet healthcare needs		All cases	Age groups			Gender		Places	
			60–69 years	70–79 years	≥ 80 years	Male cases	Female cases	Urban cases	Rural cases
Not being found after falling	N, %	625 (3.32)	174 (4.67)	226 (4.17)	225 (2.33)	370 (3.85)	255 (2.77)	86 (2.17)	539 (3.63)
	OS	0.17 (0.04, 1)	0.15 (0.04, 1)	0.13 (0.04, 1)	0.21 (0.04, 1)	0.17 (0.04, 1)	0.13 (0.04, 1)	0.29 (0.04, 1)	0.17 (0.04, 1)
Being found after falling	N, %	18,190 (96.68)	3548 (95.33)	5193 (95.83)	9449 (97.67)	9231 (9.15)	8959 (97.23)	3884 (97.83)	14,304 (96.37)
	OS	2 (0.13, 30)	0.25 (0.04, 2)	1 (0.08, 14)	7 (1, 90)	1 (0.08, 12)	4 (0.25, 90)	6 (0.33, 90)	1 (0.13, 30)
Not receiving care after falling	N, %	5649 (30.02)	1169 (31.41)	1639 (30.25)	2841 (29.37)	2956 (30.79)	2693 (29.23)	910 (22.92)	4738 (31.92)
	OS	1 (0.04, 4)	0.08 (0.04, 1)	0.17 (0.04, 2)	1 (0.08, 16)	0.21 (0.04, 2)	1 (0.08, 11)	1 (0.08, 30)	0.5 (0.04, 3)
Receiving care after falling	N, %	13,166 (69.98)	2553 (68.59)	3780 (69.75)	6833 (70.63)	6645 (69.21)	6521 (70.77)	3060 (77.08)	10,105 (68.08)
	OS	3 (0.25, 60)	1 (0.08, 4)	2 (0.13, 29.5)	12 (1, 120)	2 (0.13, 22)	7 (1, 120)	7 (1, 90)	2 (0.21, 42)

## Discussion

This study introduces a novel perspective into the understanding of the consequence of falls among older adults by investigating potential factors that may contribute to fatal outcomes. While existing literature has extensively probed into the causes of falls in elder population, our findings underscore that survivability following an accident fall is also influenced by multiple intrinsic and extrinsic factors, along with fall-related physical consequences. The global report by WHO emphasizes that falls persistently lead to high mortality rates or diminished quality of life, with a noteworthy escalation in fatal fall rates observed beyond the age of 60^11^. Falls presently rank as the second leading cause of unintentional injuries deaths, resulting in hospitalization for 37 million individuals annually due to unintentional falls, with a particular impact on those aged over 60^3,14,15^. Our study posits that the imperative lies not only in the prevention of falls among older adults but also in the effective management of ensuing injuries, thereby mitigating the growing harm, suffering, and loss, which aligns with the overarching target outlined by the Sustainable Development Goals (SDGs), aiming to ensure healthy lives and promote well-being across all ages^15^.

The study revealed that hypertension was a significant intrinsic factor associated with fatal falls, constituting the highest proportion (11.33%) among reported cases of fatal falls with medical conditions. This finding is consistent with previous studies indicating that older adults with cardiovascular disease, particularly hypertension, face an elevated risk of falls^10,16,17^. One possible explanation for this increased risk among older individuals with hypertension is the presence of gait and balance disorders. Older people often exhibit a stiffer and less coordinated gait pattern along with poorer posture control, which contributes to an augmented risk of falling^10^. Moreover, older adults with elevated blood pressure may experience heightened fall risk due to impaired gait and standing balance^10,18,19^. A recent study conducted by Abu Bakar in Malaysia also revealed that the medication for hypertension could serve as a contributing factor to falls. Older adults using diuretics as part of their polypharmacy treatment experienced a higher incidence of falls compared to non-polypharmacy users^17^, potentially attributed to the association between polypharmacy practices and poorer cognition and physical performance^20,21^.

Another revelation from our data indicates that individuals under the age of 80 were at a higher risk for fatal falls. The data pertaining to this group showed an increased susceptibility of sustaining head trauma following falls, coupled with a significantly shorter OS compared to those aged 80 and above. Previous studies have consistently demonstrated that falls often result in head injuries as opposed to injuries in other body parts^22,23^. Komisar et al.’s study reported a 42.8% probability of head injury upon impact^23^, while Tyler suggested that age-related neuromuscular changes in the neck musculature may contribute to a higher incidence of head impacts in older adults^24^. Head impacts during falls lead to elevated acceleration and greater force, which subsequently impairs head stabilization^25,26^, ultimately increasing the risk of severe traumatic brain injury and fatal falls. It is noteworthy that older individuals who acknowledge the aging process tend to adopt protective strategies; however, not all individuals over the age of 65 accept balance deterioration and instead strive for maintaining their previous capabilities^27^.

Furthermore, our findings align with previous studies indicating that fatal falls predominantly occur within the homes of older adults. Specifically, beds and bathrooms emerged as the primary locations for such incidents in senior individuals living independently, often attributed to polypharmacy and diuretic usage which were commonly observed in hypertensive older adults^28–30^. A higher proportion of fatal falls in this study occurred on stairs at home, likely due to cognitive impairment affecting seniors’ visual attention and multitasking abilities during stair descent^31^. Additionally, older adults also tend to reduce their gait and speed, alter lower limb kinematics and increase foot clearances while descending stairs^32^, leading to longer fixation times on the stairs and travel path and an increased risk of falls. In addition to the home, certain groups of seniors in our study, including those under 70 years old, males, and those living in rural areas, had a higher proportion of fatal falls at workplace. The study conducted by Roberts et al.’s proved that the labor force participation rate among adults aged 65–74 surpasses that of individuals in the 75–84 age group, with men exhibiting a higher labor force participation rate compared to women^33^. Within the younger age group, male seniors face an elevated risk of occupational falls due to their engagement in hazardous activities or tasks with high levels of difficulty, even when they experience physical health limitations^34^. While studies reported a greater propensity for falls among senior females residing in rural areas^35^, our data suggested that adverse consequences resulting from falls were severe, rendering them life-threatening events for active outdoors males. Therefore, it is crucial to consider gender-specific risk factors when designing strategies for fall prevention^36^. An unmet healthcare need normally refers to a condition in which the treatment or diagnosis is not promptly and adequately addressed by available therapeutic options. In this study, we observed that old adults who delayed or failed to receive needed care after experiencing a fall exhibited poorer health outcomes. Notably, male or rural senior adults were more likely to experience delays in receiving timely care or even no care at all, resulting in significant shorter OS compared to their counterparts. The rapid urbanization process witnessed over the past three decades has led to an influx of migrant workers into major cities, while the number of older adults living alone continues to rise in villages or small towns^37^. The phenomenon of ‘empty families’ or even ‘empty villages’ poses challenges to the traditional model of family support for older adults. Seniors living alone often face greater inconveniences, difficulties, and health risks than those residing with their adult children; thus necessitating more effective provision of care services that are currently lacking within the existing healthcare system^38^. Government agencies and institutions should prioritize community-based or home-based elderly care services tailored towards meeting the diverse needs of various groups, especially those left behind^39^.

Indeed, the majority of unintentional falls in older adults can be prevented through appropriate care or the elimination of environmental risk factors. A thorough examination of existing evidence indicates that most risk factors for falls can be identified, and approximately one to two-thirds of falls are preventable^1,40^. Even during hospitalization following a fall, most deaths resulting from falls are considered preventable or potentially preventable within healthcare settings^41^. Therefore, improving the health status of order individuals with the preventive measures is perhaps one of the cost-effective ways to optimize their functional ability. Several systematic reviews on interventions for preventing falls in older adults, including various intervention types commonly used, have found that exercise as a standalone intervention is more effective in reducing the incidence of falls and fall-related outcomes^42–44^. In our study, a significant proportion of fatal falls occurred in rural settings, particularly at workplace. Given the context of rural lifestyles where older individuals continue working in fields, falling may be attributed to increased exposure to occupational risks rather than athletic deterioration or poor mobility. In such cases, a combination of assistive technology and quality improvement strategies^43^, such as health education combined with targeted occupational protection could serve as an alternative approach to preventing future falls among rural residents. In remote rural settings where access to institutional and emergency is limited, seniors and family members should be aware of fall risk factors and potential prevention strategies. Simultaneously, village doctors should receive training on essential skills enabling them to promptly assess and provide pre-hospital acute care immediately after a fall.

Moreover, as previously mentioned, this study underscores the evident prevalence of health trauma as a significant and severe consequence of falls, particularly among individuals aged below 70. A parallel study conducted in Munich also echoed a comparable inclination towards fatal falls^45^. This finding implies the necessity to prioritize fall prevention strategies for younger age groups, involving initiatives like self-management promotion and awareness campaigns.

In addition, in the realm of fall prevention, several prudent considerations can be incorporated into government policies and national plans for addressing population aging. This is particularly crucial given the substantial impact of injurious falls on mortality and the quality of death. Firstly, it is of essentially to data collection on fall-related injuries among older adults in both rural and urban settings, particularly those living alone at home, in order to tailor interventions appropriately. Secondly, proactive measures should be implemented for the management of fall-related injury, with a focus on reducing their severity and minimizing disability, along with preventing infections following an injury^29^. Finally, at the community level, enhancing social cohesion could serve as a strategy to mitigate persistent fall-related issues^46^, and integrating palliative care components into public health services may help alleviate the suffering of older adults who have experienced severe falls, particularly those with limited access to specialized treatment.

## Limitations

The data quality of the present study relies primarily on the information available to the medical examiners or clinicians who complete the death records in the DSPs system. In order to address missing quantitative data, textual data describing the pre-death process of each fatal fall were extensively examined in this study. However, contributing factors leading to over 70% of fall-related deaths were not specified. To better describe the circumstances surrounding fall deaths, more comprehensive and detailed data should be included in the DSPs system. Training is necessary to ensure accurate completion of death report cards by medical examiners and clinicians alike. Additionally, distinguishing falls as either underlying causes or contributing causes of death can pose challenges. While a fall may initiate a chain of events resulting in death, an accompanying illness could be reported as the underlying cause instead^47^. In this study, only falls with ICD-10 codes W00-19 were considered as underlying causes of death in our analyses. Future research could explore examining and comparing deaths where falls are defined as contributing rather than underlying causes. Moreover, nonfatal falls were not included in this study. A comparative analysis of fatal and nonfatal falls may provide additional insights into preventing and managing fatal falls among older individuals.

## Conclusion

The present study systematically utilized data from DSPs to investigate the circumstances (initiating events and locations) and physiological consequences (injuries and overall survival) of fatal falls in Yunnan, China. These findings contribute to the development of strategies for preventing and managing fatal falls among older individuals. Notably, hypertension emerged as a significant intrinsic factor contributing to fatal falls among older adults with reported medical conditions. The implementation of fall risk assessment and corresponding measures, such as adjuvant therapy for cognitive or gait disorders, is crucial in modifying the risk factors to enhance the quality of life for hypertensive older adults. Individuals below 80 years old exhibited a higher incidence of fatal falls due to head injuries compared to those aged 80 or above. It is crucial to prioritize attention towards outdoor workplaces as significant sites where fatal falls occur, predominantly among rural males under 70 years old. Providing senior rural males with essential safety education or equipping them with appropriate protective gear for their involvement in agricultural production would constitute effective measures. Failure to receive timely healthcare after experiencing a fall was identified as an important factor leading to mortality in older individuals. Targeted interventions focusing on fall prevention and post-fall care are equally imperative for promoting the well-being of older adults. Additionally, considering that Yunnan province harbors the largest number of ethnic groups in China and most ethnic minorities reside in rural areas, further research on ethnic variations regarding fatal falls is essential for a comprehensive understanding in the future.

## Data Availability

The datasets generated during and/or analyzed during the current study are not publicly available due to national legislation on data security, but are definitely available from the corresponding author upon reasonable request and with permission from the Yunnan Center for Disease Control and Prevention.
